# On the Selection of Non-Invasive Methods Based on Speech Analysis Oriented to Automatic Alzheimer Disease Diagnosis

**DOI:** 10.3390/s130506730

**Published:** 2013-05-21

**Authors:** Karmele López-de-Ipiña, Jesus-Bernardino Alonso, Carlos Manuel Travieso, Jordi Solé-Casals, Harkaitz Egiraun, Marcos Faundez-Zanuy, Aitzol Ezeiza, Nora Barroso, Miriam Ecay-Torres, Pablo Martinez-Lage, Unai Martinez de Lizardui

**Affiliations:** 1 Systems Engineering and Automation Department, University of the Basque Country UPV/EHU, Donostia 20018, Spain; E-Mails: harkaitz.egiraun@ehu.es (H.E.); aitzol.ezeiza@ehu.es (A.E.); nora.barroso@ehu.es (N.B.); unai.martinezdelizarduy@ehu.es (U.M.L.); 2 Signal and Communication Departament (DSC), Institute for Technological Development and Innovation in Communications (IDeTIC), University of Las Palmas de Gran Canaria (ULPGC), Campus of Tafira, Las Palmas de Gran Canaria 35017, Spain; E-Mails: jalonso@dsc.ulpgc.es (J.-B.A.); ctravieso@dsc.ulpgc.es (C.M.T.); 3 Digital Technologies Group, University of Vic, Sagrada família 7, Vic 08500, Spain; E-Mail: jordi.sole@uvic.cat; 4 Research Center for Experimental Marine Biology and Biotechnology, Plentzia Marine Station, University of the Basque Country, Plentzia 48620, Spain; 5 Escola Universitaria Politècnica de Mataró (UPC), Tecnocampus, Mataró, Barcelona 08302, Spain; E-Mail: faundez@tecnocampus.cat; 6 CITA-Alzheimer Foundation, San Sebastian 20009, Spain; E-Mails: mecay@cita-alzheimer.org (M.E.-T.); pmlage@cita-alzheimer.org (P.M.-L.)

**Keywords:** Alzheimer's disease diagnosis, spontaneous speech, emotion recognition, machine learning, non-invasive diagnostic techniques, dementia

## Abstract

The work presented here is part of a larger study to identify novel technologies and biomarkers for early Alzheimer disease (AD) detection and it focuses on evaluating the suitability of a new approach for early AD diagnosis by non-invasive methods. The purpose is to examine in a pilot study the potential of applying intelligent algorithms to speech features obtained from suspected patients in order to contribute to the improvement of diagnosis of AD and its degree of severity. In this sense, Artificial Neural Networks (ANN) have been used for the automatic classification of the two classes (AD and control subjects). Two human issues have been analyzed for feature selection: Spontaneous Speech and Emotional Response. Not only linear features but also non-linear ones, such as Fractal Dimension, have been explored. The approach is non invasive, low cost and without any side effects. Obtained experimental results were very satisfactory and promising for early diagnosis and classification of AD patients.

## 1. Introduction

Alzheimer's disease (AD) is the most common type of dementia among elderly people in Western countries and it has a large socioeconomic cost to society which is expected to increase in the near future. It is characterized by progressive and irreversible cognitive deterioration with memory loss, impaired judgment and language and other cognitive deficits and behavioural symptoms that end up becoming severe enough to limit the ability of an individual to perform professional, social or family activities of daily life. As the disease progresses patients develop increasingly severe disabilities to finally become completely dependent. An early and accurate diagnosis of AD would be of much help for patients and their families, both to plan for the future and to start an early treatment of the symptoms of the disease.

According to current criteria, the diagnosis is expressed with different degrees of certainty as possible or probable AD when dementia is present and other possible causes have been ruled out, but an unambiguous diagnosis of AD requires the demonstration of the typical AD pathological changes in brain tissue by autopsy (*post-mortem* analysis) [1–3]. The clinical hallmark and earliest manifestation of AD is episodic memory impairment. At the time of clinical presentation other cognitive deficits are usually already present in their language, executive functions, orientation, perceptual abilities and constructional skills. Associated behavioural and psychological symptoms include apathy, irritability, depression, anxiety, delusions, hallucinations, disinhibition, aggression, aberrant motor behaviour, as well as eating or sleep behaviour changes [1–5]. All these symptoms lead to impaired performance in family, social or professional activities of daily life as the disease progresses from mild to moderate and to severe.

As already mentioned above, the diagnosis of AD is made on clinical grounds and requires, on one hand, the confirmation of a progressive dementia syndrome and, on the other, the exclusion of other potential causes of dementia by clinical history and examination, complete blood workup tests and brain-imaging analysis test, such as computer tomography (CT) or magnetic resonance imaging (MRI). The criteria to exclude other potential causes have changed in the last years as the interpretation of neuroimaging tests, including functional imaging with Single-Photon Emission Computed Tomography (SPECT) and Positron Emission Tomography (PET), has focused on the positive findings of typical AD changes, such as medial temporal atrophy detected by CT or MRI and temporoparietal hypometabolism by PET [6–9].

Nonetheless, the diagnosis of the early stages of not only mild cognitive impairment but also mild dementia remains problematic, since patients and relatives tend to either ignore the first clinical manifestations or ascribe them to the expected cognitive changes related to age. It usually takes 2 to 3 years to seek medical advice after the onset of the symptoms [1–3]. In addition, physicians may feel uncertain or uncomfortable to establish a diagnosis when the whole picture of dementia is not yet fully present; therefore, they usually feel the need to apply long neuropsychological tests, expensive neuroimaging techniques or invasive tests such as a lumbar puncture to reach a diagnosis [10]. It is consequently not surprising that most of the patients are diagnosed when they have already reached the moderate stage of the disease and have become substantially dependent. At this stage, it is very difficult for any treatment strategy to show significant efficacy to stop or even delay the disease process [2–5].

Significant advances have taken place during the last years in the early diagnosis of AD using clinical biomarkers [10], but the currently high cost and technology requirements make it unfeasible to use these diagnostic procedures on any patient displaying only memory complaints. As a result, they are usually applied to pre-selected patients based on their being highly suspect of suffering an underlying AD pathology; who are then apt to have an invasive lumbar puncture or a very expensive PET performed [10–13].

In this setting, the development of non-invasive intelligent diagnosis techniques would be very valuable for the early detection and classification of different types of dementia. Particularly, because they do not require specialized personnel or laboratory equipment, so that anyone in the habitual environment of the patient could perform (after proper training) without altering or blocking the patient's abilities [14–19]. Automatic Spontaneous Speech Analysis (ASSA) and Emotional Response Analysis (ERA) on speech are two of them [15].

Spoken language is one of the most important elements defining an individual's intellect, his/her social life and personality; it allows us to communicate with each other, share knowledge, and express our cultural and personal identity. Spoken language is the most spontaneous, natural, intuitive and efficient method of communication among people. Therefore, the analysis by automated methods of Spontaneous Speech (SS) or Automatic Speech Analysis (ASSA) which is the freer and more natural expression of communication, possibly combined with other methodologies, has the potential to become a useful non-invasive method for early AD diagnosis [15,20–24].

Emotional Response Analysis (ERA) on speech also has that potential: emotions are cognitive processes related to the architecture of the human mind, such as decision making, memory or attention, closely linked to learning and understanding that arise in intelligent systems when they become necessary to survive in a changing and partially unpredictable world [25–27]. The nonverbal information, which often includes body-language, attitudes, modulations of voice, facial expressions, etc., is essential in human communication as it has a large effect on the communication provision of the partners and on the intelligibility of speech [25]. Human emotions are affected by the environment, the direct interaction with the outside world but also by the emotional memory emerging from the experience of individual and cultural environment, the so called socialized emotion. Emotions use the same components subjective, cultural, physiological and behavioural that the individual's perception expresses with regard to the mental state, the body and how it interacts with the environment [26,27]. In this work ERA has been analyzed by classical features and by Emotional Temperature, described in Section 3. This feature is based on the analysis of several prosodic and paralinguistic features sets obtained from a temporal segmentation of the speech signal.

Finally, we wished to apply non-invasive methods to estimate the severity of Alzheimer in the patient. In this sense, analysis of spontaneous speech is not perceived as a stressful test and moreover its cost is lower than for other methods. None of these speech analysis based techniques require extensive infrastructure or the availability of medical equipment, and suggest obtaining by these means is easy, quick and inexpensive [14,15].

**Figure 1.**
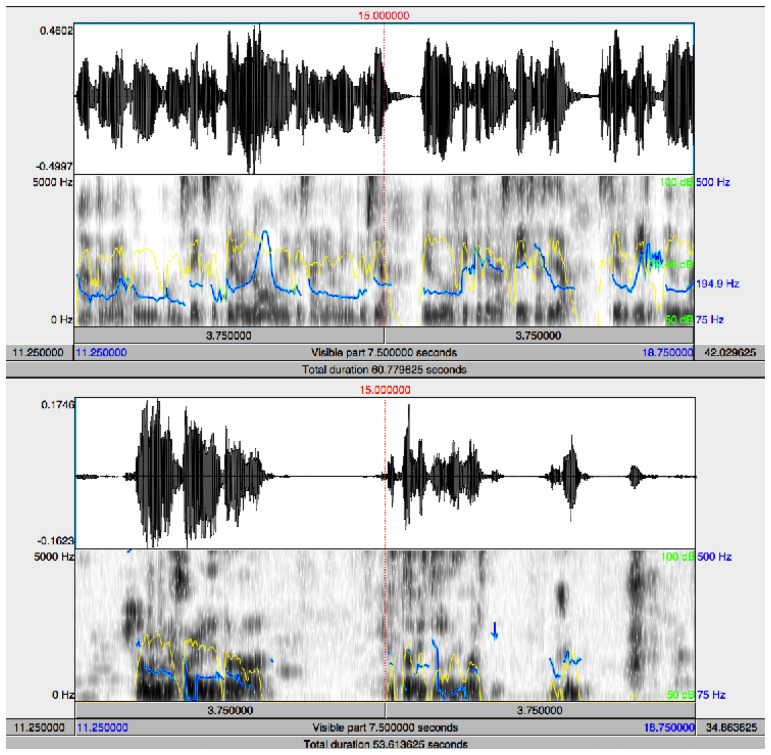
Signal and spectrogram of a control subject (**Top**) and an AD subject (**Bottom**) during spontaneous speech (pitch in blue, intensity in yellow).

We have focused our work on non-invasive diagnostic techniques based on the analysis of speech and emotions because after the loss of memory, one of the major problems of AD is the loss of language skills, illustrated by the poorer signal and spectrogram during spontaneous speech of the AD patient shown in Figure 1. This loss is reflected in difficulties both to speak and to understand others, which makes more difficult the natural communication process with the environment. The inability to communicate appears already in the early phases of the diseases. It is possible to find different communication deficits in the area of language, including [28,29] aphasia (difficulty in speaking and understanding) and anomic aphasia (difficulty for recognizing and naming things). The specific communication problems the patient encounters depend on the stage of the disease [2–5,28,29]:

1. *First Stage or early stage (ES)*: difficulty in finding the right word in spontaneous speech. Often remains undetected.
2. *Second Stage or intermediate stage (IS)*: impoverishment of language and vocabulary in everyday use.
3. *Third Stage or advanced stage (AS)*: answers sometimes very limited and restricted to very few words.

Not only the language but also the emotional responses in Alzheimer's patients become impaired and seem to go through different stages. In the early stages, social and even sexual disinhibition appears and behavioural changes are also observed (for example, being angry and not being able to perform common tasks, express themselves or remember) [30–33]. However, the emotional memory remains, and they cry more easily and gratefully acknowledge caresses, smiles and hugs. The Alzheimer's patient reacts aggressively to things that for healthy people are harmless, and perceives a threat or danger where none exists. In more advanced stages they may often seem shy and apathetic, symptoms often attributed to memory loss and/or difficulty in finding the right words and some responses are likely to be magnified due to an alteration in perception.

Alternatively, it has been suggested that the reduced ability to feel emotions is due to memory loss, which may in turn induce the appearance of apathy and depression [31,33]. The work presented here is part of a larger study to identify novel technologies and biomarkers for early AD detection, and it focuses on evaluating the suitability of a new approach for early AD diagnosis based on non-invasive and low cost methods, namely Automatic Spontaneous Speech Analysis and the Emotional Response Analysis, whose results are susceptible to be used for the automatic classification of tested individuals.

## 2. Materials

### 2.1. Main Database of Individuals

Trying to develop a new methodology applicable to a wide range of individuals of different sex, age, language and cultural and social background, we have built up a multicultural and multilingual (English, French, Spanish, Catalan, Basque, Chinese, Arabian and Portuguese) database with the video recordings of 50 healthy and 20 AD patients (with a previous diagnosis) recorded for 12 hours and 8 hours respectively. The age span of the individuals in the database was 20–98 years and there were 20 males and 20 females. This database is called AZTIAHO.

All the work was performed strictly following the ethical consideration of the organizations involved in the project. The recordings consisted of videos of Spontaneous Speech where people tell pleasant stories or feelings and interact with each other in a friendly conversation. The recording atmosphere is relaxed and non-invasive. The shorter recording times for the AD group are due to the fact that AD patients find speech more of an effort than healthy individuals: they speak more slowly, with longer pauses, and with more time spent on efforts to find the correct word and uttering speech disfluencies or break messages. In the advanced stage of the disease, they find this effort tiring and often want to stop the recording. We complied with their requests.

### 2.2. Pre-Processing

Video has been processing and audio extracted in wav format (16 bits and 16 kHz). Firstly non-analyzable events have been removed: laughter, cough, short hard noises and speaker mixes. Then background noise has been removed by a denoiser adaptive filtering. After the pre-processing, about 80% and 50% of the material from the control and AD groups respectively, is suitable for further analysis. The full database consisted of about 60 minutes for the AD group and of about 9 hours for the control. The speech is divided into consecutive segments of 60 seconds in order to obtain appropriate segments for all speakers. Finally, a database of about 600 segments of spontaneous speech is obtained.

### 2.3. Individuals Selected for the Study

From the original database, a subset of 20 AD patients (68–96 years of age, 12 women, 8 men, within the three stages of AD: First Stage (ES = 4), Secondary Stage (IS = 10) and Tertiary stage (AS = 6) was chosen. The control group (CR) was made up of 20 individuals (10 male and 10 female, aged 20–98 years) representing a wide range of speech responses. This subset of the database is called AZTIAHORE.

## 3. Methods

### 3.1. Feature Extraction

#### 3.1.1. Automatic Spontaneous Speech Analysis (ASSA)

The analysis of spontaneous speech fluency is based on three families of features (SSF set), obtained using Praat software [34]. For that purpose, an automatic Voice Activity Detector (VAD) [35,36] has extracted voiced/unvoiced segments, as parts of an acoustic signal. These three families of features include:

1. *Duration:* this includes the histogram calculated over the most relevant voiced and unvoiced segments, the average of the most relevant voiced/unvoiced, voiced/unvoiced percentage and spontaneous speech evolution along the time, and the voiced and unvoiced segments' mean, max and min.
2. *Time domain:* short time energy.
3. *Frequency domain, quality:* spectral centroid.

**Figure 2.**
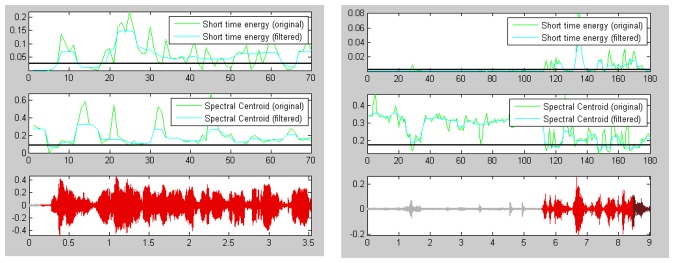
Plots of speech signal, Short Time Energy and Spectral Centroid, all measures filtered by a median filter, for a control subject (**Left**) and an AD subject (**Right**).

The energy of a signal is typically calculated on a short-time basis, by windowing the signal at a particular time, squaring the samples and taking the average. The spectral centroid is commonly associated with the measure of the brightness of a sound. This measure is obtained by evaluating the “centre of gravity” using the Fourier transform's frequency and magnitude information (Figure 2).

#### 3.1.2. Higuchi Fractal Dimension

When appropriate corpora are available, linear systems can be implemented fairly rapidly, as they rely on well-known machine learning techniques to achieve their goals, avoiding complex adjustments to the system. These latter types of tasks often require experimentation with alternative techniques, which can lead to improved systems. One such alternative technique of particular interest is nonlinear analysis, and some works show that combining nonlinear features with linear ones can produce higher recognition accuracies without substituting the whole linear system with novel nonlinear approaches (see [37,38] for examples on nonlinear speech processing). This is especially promising for solving non-typical tasks, since it would be very demanding to design a complete nonlinear system from scratch for solving a task already made difficult by the scarcity of resources.

**Figure 3.**
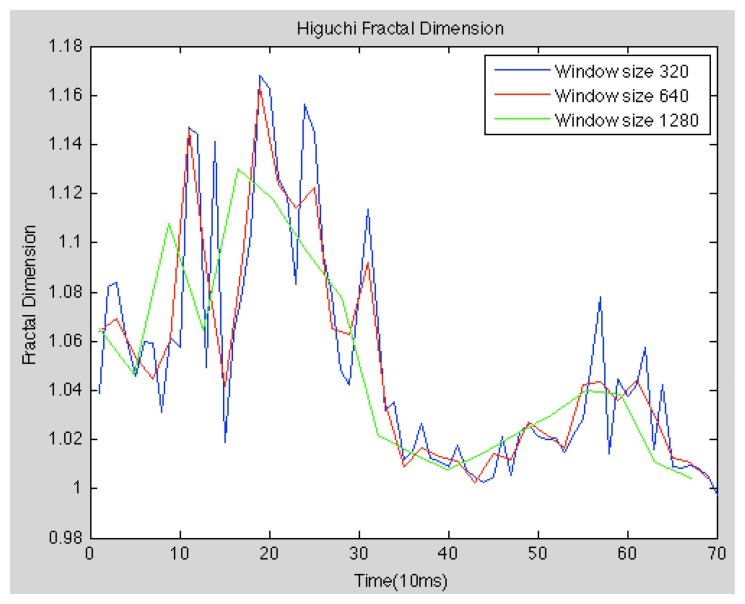
Higuchi Fractal Dimension *c* of speech signal for an AD subject and different window length.

The fractal dimension is one of the most popular features, which describe the complexity of a system. Most if not all of the fractal systems have a characteristic called self-similarity. An object is self-similar if a close-up examination of the object reveals that it is composed of smaller versions of itself. Self-similarity can be quantified as a relative measure of the number of basic building blocks that form a pattern, and this measure is defined as the fractal dimension. This current work focus on the alternatives, which do not need previous modelling of the system. Higuchi proposed an algorithm for measuring the fractal dimension of discrete time sequences directly from time series, so in our experiments we use the method described in [39] (see Figure 3).

#### 3.1.3. Emotional Speech Analysis (ESA)

In this study we aim to accomplish the automatic selection of emotional speech by analyzing three families of features in speech:

1. *Acoustic features*: pitch, standard deviation of pitch, max and min pitch, intensity, standard deviation of intensity, max and min intensity, period mean, period standard deviation, and Root Mean Square amplitude (RMS);
2. *Voice quality features*: shimmer, local jitter, Noise-to-Harmonics Ratio (NHR), Harmonics-to-Noise Ratio (HNR) and autocorrelation;
3. *Duration features*: fraction of locally unvoiced frames, degree of voice breaks.

Short-term energy is the principal and most natural feature that has been used. Physically, energy is a measure of how much signal exists at any one time. Energy is used in a continuous speech to discover voiced sounds, which have higher energy than silence/un-voiced, as shown in Figure 2.

The energy of a signal is typically calculated on a short-time basis, by windowing the signal at a particular time, squaring the samples and taking the average [36]. The square root of this result is the engineering quantity, known as the root-mean square (RMS) value.

#### 3.1.4. Emotional Temperature

The Emotional Temperature (ET) is based on the analysis of a few prosodic and paralinguistic features sets obtained from a temporal segmentation of the speech signal [40–42].

Two prosodic and four paralinguistic features related to the pitch and energy, respectively, were estimated from each frame. These features were chosen because their robustness in emotion recognition has been proven [43–47], they are quickly and easily calculated, and they are independent of linguistic segmentation, which helps us to avoid problems in real time applications on real environments. For prosodic features, a voiced/unvoiced decision is made to each frame and two linear regression coefficients of the pitch contour [43–46] are obtained. For paralinguistic features, voice spectral energy balances [43,47] are calculated from each frame, and quantified using 4 percentages of energy concentration in 4 frequency bands. Then Emotional Temperature is calculated as follows:

1. A Support Vector Machine (SVM) is trained with a balanced segment set extracted from the database (SVMs have been used to quantify the discriminative ability of the proposed measures [45]. We have used a freely available implementation called LIBSVM [48] with a radial basis kernel function).
2. For each speech segment, each temporal frame is classified by the SVM as “pathological or “non-pathological”.
3. The percentage of temporal frames classified as “non-pathological” is calculated. This value, *i.e.*, the number of non-pathological frames is the “emotional temperature”.
4. The “Emotional Temperature” is finally normalized in order to have ET = 50 as threshold obtained from the training database, which indicates the limit between pathological and non-pathological frames. This normalization will help medical specialists to easily interpret the data.

**Figure 4.**
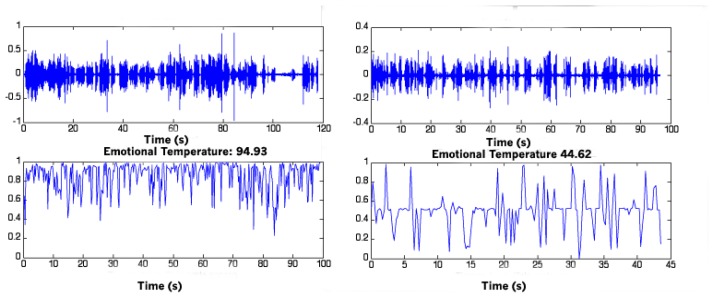
Emotional Temperature for a healthy subject (**Left**) and an AD subject (**Right**).

Figure 4 shows an example of ET values for a healthy subject (ET = 94.93) and for an AD subject (ET = 44.62).

#### 3.1.5. Feature Sets

Based in these presented characteristics, four feature sets have been created for experimentation:

1. SSF.
2. EF, set described in Section 3.1.3.
3. FD1: Higuchi Fractal Dimension (HFD).
4. FD2: HFD, maximum HFD, minimum HFD, variance HFD and standard deviation HFD.

### 3.2. Automatic Classification

The automatic classification of emotional speech is based on the Multi Layer Perceptron (MLP) neural network with one hidden layer of 100 neurons and 1,000 training steps. WEKA [49] software has been used in carrying out the experiments. The results are evaluated using Accuracy (Acc) and Classification Error Rate (CER) measurements. For the training and validation steps, we used k-fold cross-validation in order to ensure solid results. Cross validation is a robust validation for variable selection [50]. These features will define CR group and the three AD levels. The original sample set was randomly divided into k subsets. Then, a single subset was retained as the validation data set for testing the model, and the remaining k-1 subsets were used as training data. The cross-validation process was repeated k times, with each of the k subsets used exactly once as the validation data set. The k obtained values from the folds, were then averaged to obtain the final result. The advantage of this method is that all observations are used for both training and validation, and each observation is used for validation exactly once. In our experiments we use k = 10. These features will discriminate among control group (CR) and the three AD levels.

## 4. Results and Discussion

The experimentation has been carried out with the balanced subset AZTIAHORE. The goal of these experiments was to examine the potential of selected features for automatic measurement of the degradation of Spontaneous Speech, Emotional Response and their integration in people with AD. Thus, previously defined feature sets have been evaluated in order to properly define control and AD level groups.

In a first stage Emotional Temperature is calculated for each segment by the method described in Section 3.1.4. Automatic classification by MLP was performed over the speech features sets described in Section 3.1.5 in order to analyze for the pilot study the tests: Automatic Spontaneous Speech fluency, Emotional Response in speech and both in Integral Speech. Table 1 summarized Accuracy (%) global results with regard to pre-clinical test and feature sets.

- In the first test, *Spontaneous Speech Fluency* test, SSF set alone has been used and also integrated with two different Fractal Dimension (FD) sets. The use of FD features outperforms the results, and the system obtains an improvement of %10 with FD2 set, which includes several FD features.
- In the second test, *Emotional Response* test, the best result is obtained when ET feature is included being Acc near to the optimum value.
- With regard to the *INTEGRAL* test, (which includes information relative to both Spontaneous Speech and Emotional Response) results show also an improvement of non-linear features and better results when ET is included.

**Figure 5.**
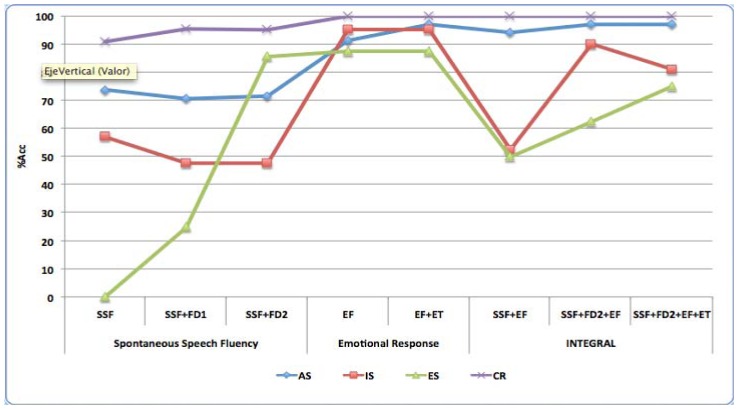
Accuracy in % for the three defined tests in the pilot study and each corresponding feature sets.

Figure 5 shows the obtained results for control group (CR) and the three levels of AD (ES, IS and AS) for classes. In these classes' results the inclusion of non-linear features FD2 set obtains the best results for all classes (Figure 5). This set improves also the classification with regard to early detection (ES class). IS has also better rate to discriminate middle AD level. The model is able also to discriminate pathological and non-pathological segments in each patient for the three tests of the pilot study.

**Figure 6.**
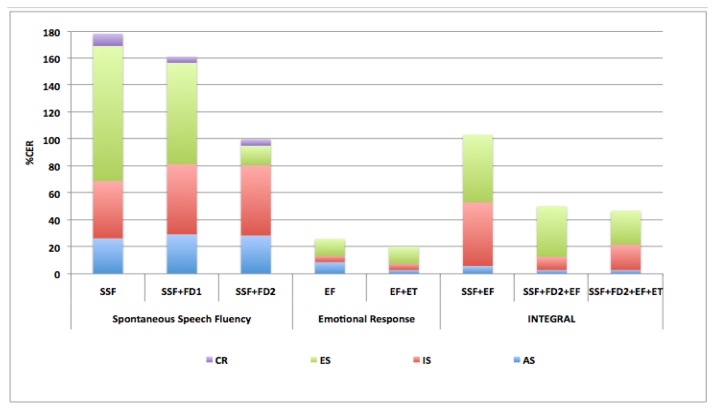
Accumulative Classification Error Rate (%) for the three defined tests and each corresponding feature sets.

Accumulative Classification Error Rate (%CER) is detailed in Figure 6. Less accumulative error rate is obtained for experiments which include FD2 non-linear feature set and ET. This is relevant for early diagnosis because in these cases better results are obtained with smaller classification error for ED class.

**Figure 7.**
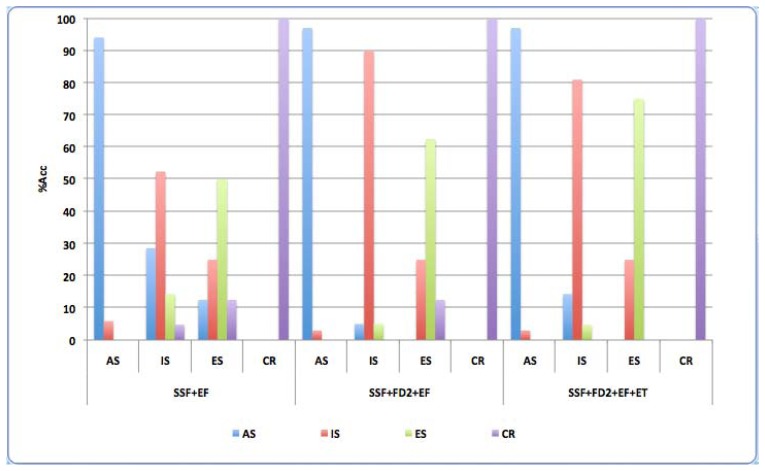
Accuracy (%) of classes for *INTEGRAL* test and each corresponding feature sets.

A detailed analysis of *INTEGRAL* test, with regard to Acc (%) for all classes is showed in Figure 7.

- When the results with simple speech features are analyzed, it is observed optimum performance for control group but very mixed classification in AD levels mainly for IS and ES.
- The global system obtains very good results when FD2 set is added because not only outperforms Acc in middle stage IS, but also there is a great improvement in Acc for ES.
- In the third experiment, ET is includes and Acc obtains very good results for all classes, and ES outperforms because confusion disappears with regard to the segments of control subjects.

Finally, the health specialists notice the relevance of the system's ability to carry out both the analysis of independent biomarkers as Spontaneous Speech and Emotional Response features, and/or the integral analysis of several biomarkers. The final confusion among segments could be due, in some cases, to the possible occurrence of segments with different pathological levels in the same individual. This possibility will be explored with new tests in future works. Therefore these non-invasive tests could be a very useful tool for medical specialists' in future clinical AD early diagnosis grounds.

## 5. Conclusions

The main goal of the present work is the analysis of features in Spontaneous Speech and Emotional Response oriented to pre-clinical evaluation for the definition of appropriate tests for early AD diagnosis. These features are of great relevance for health specialists to define health people and the three AD levels. Features relative to speech duration, time domain, spectral domain and fractal dimension have been analyzed. In this work a first approach including nonlinear features is described. More precisely, an implementation of Higuchi's algorithm in order to add this new feature to the set that feeds the training process of the model. The approach's performance is very satisfactory and promising results for early diagnosis and classification of AD patient groups. Moreover, new features (Higuchi Fractal Dimension and Emotional Temperature) significantly outperform previous results. In future work we will evaluate this approach with an early diagnosis database and new tests oriented to semantic and memory tasks, and we will also introduce new features relatives to non-linear dynamic.

## Figures and Tables

**Table 1.** Accuracy (%), global results with regard to test and feature sets.

**Test**	**Feature Set**	**%Acc**
**Spontaneous Speech Fluency**	SSF	75.2
	SSF + FD1	76.7
	SSF + FD2	86.1
**Emotional Response**	EF	90.7
	EF + TE	97.7
**INTEGRAL**	SSF + EF	92.2
	SSF + FD2 + EF	94.6
	SSF + FD2 + EF + TE	94.6
